# Health-related quality of life in patients waiting for major joint replacement. A comparison between patients and population controls

**DOI:** 10.1186/1477-7525-4-3

**Published:** 2006-01-19

**Authors:** Johanna Hirvonen, Marja Blom, Ulla Tuominen, Seppo Seitsalo, Matti Lehto, Pekka Paavolainen, Kalevi Hietaniemi, Pekka Rissanen, Harri Sintonen

**Affiliations:** 1 National Research and Development Centre for Welfare and Health, Helsinki, Finland; 2 University of Helsinki, Finland; 3 Academy of Finland; 4 HUCH, Jorvi Hospital, Espoo, Finland; 5 Orton Orthopaedic Hospital, Helsinki, Finland; 6 Coxa, Hospital for Joint Replacement, Medical Research Fund of Tampere University Hospital, Finland; 7 HUCH, Surgical Hospital, Helsinki, Finland; 8 University of Tampere, Finland

## Abstract

**Background:**

Several quality-of-life studies in patients awaiting major joint replacement have focused on the outcomes of surgery. Interest in examining patients on the elective waiting list has increased since the beginning of 2000. We assessed health-related quality of life (HRQoL) in patients waiting for total hip (THR) or knee (TKR) replacement in three Finnish hospitals, and compared patients' HRQoL with that of population controls.

**Methods:**

A total of 133 patients awaiting major joint replacement due to osteoarthritis (OA) of the hip or knee joint were prospectively followed from the time the patient was placed on the waiting list to hospital admission. A sample of controls matched by age, gender, housing and home municipality was drawn from the computerised population register. HRQoL was measured by the generic 15D instrument. Differences between patients and the population controls were tested by the independent samples t-test and between the measurement points by the paired samples t-test. A linear regression model was used to explain the variance in the 15D score at admission.

**Results:**

At baseline, 15D scores were significantly different between patients and the population controls. Compared with the population controls, patients were worse off on the dimensions of moving (P < 0.001), sleeping (P < 0.001), sexual activity (P < 0.001), vitality (P < 0.001), usual activities (P < 0.001) and discomfort and symptoms (P < 0.001). Further, psychological factors – depression (P < 0.001) and distress (P = 0.004) – were worse among patients than population controls. The patients showed statistically significantly improved average scores at admission on the dimensions of moving (P = 0.026), sleeping (P = 0.004) and discomfort and symptoms (P = 0.041), but not in the overall 15D score compared with the baseline. In patients, 15D score at baseline (P < 0.001) and body mass index (BMI) (P = 0.020) had an independent effect on patients' 15D score at hospital admission.

**Conclusion:**

Although patients' HRQoL did not deteriorate while waiting, a consistently worse HRQoL was observed in patients waiting for major joint replacement compared with population controls.

## Background

The OECD Waiting Times project [1] on waiting time variations for elective surgery across OECD country showed waiting times to be "a significant health policy concern" in almost half of all OECD countries. Finland and the United Kingdom were the countries with the highest waiting times.

In Finland, major joint replacements are surgical procedures with high volume and relatively long waiting times. In 2003, almost 8 800 hip replacement patients (169 per 100 000) and 6 800 knee replacement patients (131 per 100 000) were operated in Finnish hospitalss [2]. Between 1987 and 2002, the THR rate rose on average by 5% annually and the TKR rate by 12% [3]. Comparing waiting times among Finnish THR and TKR patients shows significant regional differences and a trend towards longer waiting times within the last ten years. In 2003, for patients with primary THR, the median waiting time was 155 days, and for patients with TKR 205 days [2].

To ensure the availability of care in Finland, the Council of State initiated in 2001 a national project to secure the future of health care. Guidelines for the implementation of a nationwide system for assessing health care needs and for the treatment criteria were prepared by the end of 2003. The national principles of access to hospital treatment within six months or less came into force in March 2005.

Several studies have assessed health-related quality of life (HRQoL) among patients who have undergone major joint replacement and shown that patients experience substantially more pain and restrictions in physical function than the general population [4-6]. Still, relatively few of them have examined the change in HRQoL that occurs while waiting for surgery. Studies have mostly focused on the outcomes of surgery, reported improvements in physical function, vitality and mental health and reductions in pain, or have shown that total knee arthroplasty (TKA) and total hip arthroplasty (THA) are beneficial and effective [7-13]. However, the interest in examining the relationship between HRQoL and time spent waiting for surgery has been on the increase since the beginning of 2000. The results have shown no consistent evidence that HRQoL is worse in patients having to wait longer [14-16]. However, a prospective Canadian study concluded that patients who wait 6 months at the most realize greater gains in HRQoL than those waiting longer [17]. Further, a prospective study of patients waiting for total hip arthroplasty (THA) found that patients in a later phase of disease did not reach the same level after THA as those with better preoperative function [4].

Although the principle of equal access to surgeries and other health services has been promoted by health policy in many western health care systems, practices do not totally equate to policy targets. A common view is that delayed access to care may impose a variety of costs such as welfare losses during the waiting period [18]. Still, evidence of the effect of waiting on patients' health status is mixed.

The purpose of this study is to assess HRQoL in patients awaiting major joint replacement and to compare the HRQoL of patients with that of population controls. The data collected for this analysis is part of long-term follow-up data for patients in a prospective multi-centre study aimed at assessing the costs and effects of waiting.

## Methods

### Data collecting

Patients were enrolled into this study in three Finnish hospitals (HUCH Surgical Hospital, Helsinki; HUCH Jorvi Hospital, Espoo and Coxa Hospital for Joint Replacement, Tampere) in two hospital districts (Hospital District of Helsinki and Uusimaa and Pirkanmaa Hospital District). Two hospitals provide surgical services for municipalities in the capital area. The third hospital is specialised in endoprosthetic surgery which provides services for municipalities, local and central hospitals, as well as for patients paying the costs themselves.

Patients were recruited into the study through regular contact with the orthopaedic surgeons and practice staff. The *Ad hoc *recruitment began in August 2002 and finished in November 2003.

The inclusion criteria were: need for a primary total joint arthroplasty due to osteoarthritis (OA) of the hip or knee joint (excluding rheumatoid arthritis, fractures, haemophilia and deformity) as evaluated by the hospital surgeon, a patient aged 16 years or older was placed on the waiting list in a research hospital, and the patient was willing and mentally able to participate in the study. Each patient provided a signed informed consent. The study had ethical approval from the Helsinki University Central Hospital (HUCH) Surgery Ethics Committee.

Patients completed a self-administered questionnaire at two specific points in time: 1) when placed on the waiting list (baseline), and 2) at hospital admission. The questionnaires were distributed to patients at hospital. Return of the questionnaires was via postal means. Common guidelines for administering the questionnaires were provided at each hospital.

For each patient, two population controls matched by age, gender, housing (living alone vs. living with someone) and home municipality were obtained from the National Population Register of Finland. To minimise the loss of participants, two controls per patient were selected. Thus control subject who did not return the questionnaire was replaced with the other control of the same patient. In the autumn of 2003, controls were mailed a self-administered questionnaire similar to the patients' questionnaire.

### HRQoL instrument

We assessed HRQoL using 15D. The 15D is a generic and standardised HRQoL instrument consisting of 15 dimensions: moving, seeing, hearing, breathing, sleeping, eating, speech, elimination, usual activities, mental function, discomfort and symptoms, depression, distress, vitality and sexual activity. For each dimension, the respondent must choose one of the five levels that best describes his/her state of health at the moment (best level = 1; worst level = 5) [19,20]. The single index (15D score) on a 0–1 scale, representing the overall HRQoL, is calculated from the health state descriptive system by using a set of population-based preference or utility weights. Such a weight for each level of each dimension is obtained by multiplying the level value by the importance weight of the dimension at that level [21]. The level values on a 0–1 scale, reflecting the goodness of the levels relative to no problems on the dimension (= 1) and to being dead (= 0), and the importance weights summing up to unity, have been elicited from representative population samples. The 15D has been/is being utilised among different patient groups (e.g. patients undergoing hip or knee arthroplasty) to assess outcomes from health care interventions [13,20,22]. In most of the important properties (eg. responsiveness, sensitivity, reliability and validity), the 15D compares favourably with other instruments of the same kind, such as EQ-5D, HUI3, SF-6D and AQoL [20,21,23-25].

The interpretation on the minimum clinically important difference in the 15D score is a difference ± 0.03 or more (on a scale 0–1) in the sense that people can feel the difference in health status [26].

### Statistical analysis

Data were analysed using SPSS for Windows, version 12.0.1. Descriptive statistics were used to describe demographic characteristics. Comparative analyses of demographic characteristics between patients and population controls were computed using either the independent samples t-test or the Chi-squared test depending on the levels of measurement.

Univariate analyses were conducted to determine a) the differences in the 15D score and dimensions between patients and population controls, and b) the differences between the baseline and admission measurements within the patient group. Mean group scores were compared using the paired samples t-test test within the patient group, and independent samples t-test between patients and population controls. Two-sided P-values were calculated in all tests. A P-value < 0.05 was considered statistically significant.

A multiple linear regression (MLR) model on the patient data was constructed to determine the relationships between the independent variables (waiting time, BMI, affected joint, 15D score at baseline, gender, age, education, housing) and 15D score at admission. Waiting time was skewed and thus included in the model as a categorical variable (over 3–6 months, over 6 months and 0–3 months as a reference level). All available independent variables were included in the model. The results are presented in the form of unstandardised β-coefficients.

Missing values for the 15D dimensions were predicted with the responses on the other dimensions, age and gender as explanatory variables [19]. The missing value was substituted if a minimum 80% of dimensions were present.

## Results

### Patient and population controls characteristics

Of the 197 eligible patients recruited into the study, 30 were excluded because their controls declined to participate. In addition, 6 patients did not complete the baseline and 28 did not complete the admission questionnaire and were excluded. The analysis presented here focuses on 133 pairs with completed questionnaires.

The average age of the study population including patients and age matched population controls was 67.6 years (range, 36–86 years) (Table 1). Of patients, 73 (55%) were waiting for primary THR and 60 (45%) were waiting for primary TKR. The majority (54%, n = 143) of the participants (including patients and population controls) were from capital area. A total of 75 (28%) participants were from other urban area and 48 (18%) from rural area.

**Table 1:** Characteristics of patients and population controls

**Characteristic**	**Patients n = 133**	**Population controls n = 129–133**^b^	**Patients excluded n = 61–64**^b^	**P value**^c^	**P value**^d^
Age, years (mean ± SD)	67.6 (8.8)	67.6 (8.8)	66.0 (13.2)	1.000	0.375
Females [n, (%)]	83 (62.4)	81 (60.9)	43 (67.2)	0.801	0.513
Home municipality [n, (%)]				0.900	0.534
Capital area	72 (54.1)	71 (53.4)	38 (59.4)		
Other urban area	36 (27.1)	39 (29.3)	18 (28.1)		
Rural area	25 (18.8)	23 (17.3)	8 (12.5)		
Housing, living alone [n, (%)]	39 (29.3)	40 (30.3)	29 (47.5)	0.862	0.014
Professional examination, yes [n, (%)]	45 (33.8)	61 (47.3)	23 (37.7)	0.027	0.600
Employment status [n, (%)]				0.229	0.066
Employed	17 (12.8)	27 (20.3)	16 (26.2)		
Retired	112 (84.2)	101 (75.9)	43 (70.5)		
Other	4 (3.0)	5 (3.8)	2 (3.3)		
Comorbidity, yes [n, (%)]	89 (66.9)	98 (73.7)	48 (78.7)	0.227	0.095
BMI^a ^(mean ± SD)	29.0 (4.4)	26.8 (4.4)	28.3 (4.7)	<0.001	0.280
Waiting time, days [Md, range]	71 (8–600)				
Months waiting for surgery [n, (%)]					
0–3 months	94 (70.7)				
> 3–6 months	20 (15.0)				
> 6 months	19 (14.3)				

^a ^BMI, body mass index (wt/ht^2^)

^b ^Number of observations varies due to missing values.

^c ^Between patients and population controls

^d ^Between the patients who completed the questionnaires (baseline and admission, n = 133) and those excluded (n = 64)

A comparison between patients and population controls showed that controls had more often professional education than patients and patients were heavier than controls. Of patients, 21 (16%) had a normal BMI (<25) and 112 (84%) were overweight or obese (BMI ≥ 25). Of population controls, 45 (34%) had a normal BMI, and 86 (66%) were overweight or obese.

For the patients, the waiting time from the surgeon appointment to the surgery was skewed such that a total of 94 (71%) patients waited for surgery 0–3 months, 20 (15%) waited > 3–6 months and 19 (14%) waited over 6 months. Two patients waited over one year.

A comparison between patients who completed the questionnaires (baseline and admission) and those who were excluded showed that those who were excluded were more often living alone than the completers (X^2 ^= 6.1, P = 0.014). There was, however, no statistically significant or clinically important difference in the baseline 15D score between the completers and those excluded (0.778 and 0.777, respectively; Δ0.001, t = 0.03, P = 0.980).

### HRQoL among patients and population controls

At the time the patients were placed on the waiting list, the average (SD) 15D score was 0.778 (0.091) (Table 2). Among the population controls, the mean (SD) 15D score was 0.883 (0.103). The difference was statistically significant and clinically important. The difference between the groups remained statistically significant and clinically important when patients' HRQoL at admission was compared with the HRQoL among the population controls. At baseline, patients had statistically significantly lower scores on the dimensions of moving, sleeping, usual activities, discomfort and symptoms, depression, distress, vitality and sexual activity compared to population controls.

**Table 2:** The average 15D scores and dimension level values between patients and population controls

**Health outcome**	**Patients**	**Population controls**	**Mean difference**^b ^**(95% CI)**
**15D dimension**^a^			
Moving	0.565 (0.127)	0.883 (0.172)	0.317*** (0.281, 0.354)
Seeing	0.909 (0.176)	0.943 (0.140)	0.034ns (-0.004, 0.073)
Hearing	0.914 (0.142)	0.941 (0.143)	0.027ns (-0.007, 0.062)
Breathing	0.866 (0.204)	0.867 (0.219)	0.001ns (-0.050, 0.052)
Sleeping	0.685 (0.224)	0.803 (0.186)	0.117*** (0.068, 0.167)
Eating	0.992 (0.053)	0.992 (0.053)	0.000ns (-0.013, 0.013)
Speech	0.989 (0.057)	0.978 (0.079)	-0.011ns (-0.028, 0.005)
Elimination	0.848 (0.202)	0.876 (0.193)	0.028ns (-0.020, 0.076)
Usual activities	0.655 (0.217)	0.870 (0.199)	0.214*** (0.164, 0.264)
Mental function	0.864 (0.178)	0.897 (0.170)	0.033ns (-0.009, 0.075)
Discomfort and symptoms	0.473 (0.236)	0.784 (0.204)	0.311*** (0.258, 0.364)
Depression	0.829 (0.177)	0.902 (0.138)	0.074*** (0.035, 0.112)
Distress	0.831 (0.188)	0.892 (0.155)	0.061** (0.019, 0.102)
Vitality	0.748 (0.172)	0.852 (0.152)	0.104*** (0.065, 0.143)
Sexual activity	0.731 (0.273)	0.869 (0.239)	0.138*** (0.076, 0.200)
**15D score**	0.778 (0.091)	0.883 (0.103)	0.105*** (0.082, 0.129)

n = 133

^a ^Data are mean (SD) scores. The scale is 0–1, worst to best.

^b ^Baseline scores between patients and population controls. Positive difference indicates better score and negative difference indicates worse score for population controls than for patients. ns, non-significance

* P < 0.05, ** P < 0.01, *** P < 0.001

### Change in patients' HRQoL while waiting

In patients, the 15D score improved while waiting, but the change was not statistically significant or clinically important (Δ0.008, t = 1.6, P = 0.123, 95% confidence interval, CI: 0.002–0.019). The patients showed, however, statistically significantly improved average scores at admission for moving (Δ0.032, t = 2.2, P = 0.026, 95% CI: 0.004–0.060), sleeping (Δ0.042, t = 3.0, P = 0.004, 95% CI: 0.014–0.071) and discomfort and symptoms (Δ0.038, t = 2.1, P = 0.041, 95% CI: 0.002–0.075) compared with the baseline measurement (not shown).

### Patients' HRQoL at admission

The results of the MLR analysis indicated that BMI (β = -0.003, P = 0.020) and the 15D score at baseline (β = 0.752, P < 0.001) were significantly associated with the 15D at admission (Table 3). A higher BMI when placed on the waiting list was associated with the worse 15D score at admission and the higher 15D score at baseline was associated with higher HRQoL at admission. The length of waiting was unrelated to the 15D score at admission.

**Table 3:** Multiple linear regression coefficient estimates for the patients' 15D score at admission

**Explanatory variables**	**β**^a^	**95% CI for β**	**P value**
Waiting time			
0–3 months	Reference		
> 3–6 months	0.013	-0.016, 0.043	0.381
> 6 months	0.017	-0.014, 0.047	0.286
BMI	-0.003	-0.005, -0.0004	0.020
Affected joint (0 = hip, 1 = knee)	0.013	-0.009, 0.035	0.232
15D score at baseline	0.752	0.637, 0.867	<0.001
Gender (0 = female, 1 = male)	-0.008	-0.032, 0.015	0.479
Age	-0.0004	-0.002, 0.001	0.539
Professional education (0 = no, 1 = yes)	-0.0003	-0.023, 0.022	0.976
Housing (0 = living alone, 1 = living with someone)	0.009	-0.016, 0.034	0.469
Constant	0.301		
R square	0.613		
F	21.430***		
n	133		

A positive value indicates improvement in the 15D score, and a negative value indicates worsening.

^a ^multivariate unstandardised linear regression coefficient

*** P < 0.001

## Discussion

The aim of this multi-centre study was to assess HRQoL in patients awaiting major joint replacement and to compare the HRQoL of patients with that of population controls. Patients were recruited into the study in three large Finnish hospitals across two hospital districts and were prospectively followed from the time the patient was placed on the waiting list to the time of admission, with waiting times calculated exactly. HRQoL was measured by the 15D, which is a generic, standardised, self-administered measure and has been utilised in clinical economic evaluations and population studies [20].

Some previous studies have reported that those awaiting hip or knee replacement have a significantly poorer quality of life – especially in physical and social life – than a general population [5,27]. The results of this study are in line with those studies. Our first main finding was that at both measurement points, patients awaiting major joint replacement suffered from a significantly poorer HRQoL – especially in moving, sleeping, usual activities, discomfort and symptoms, depression, distress, vitality and sexual activity – compared to the population controls. However, mental function seemed unaffected by the disease. This finding seems to be in line with an English case-control study of patients awaiting hip replacement for osteoarthritis [5], but in contrast to a recent Australian study by Ackerman et al. [28] who found that patients waiting for joint replacement suffered significantly higher psychological distress compared with the general population.

Our second main finding was that patients' overall HRQoL improved while waiting although the improvement was not statistically significant or clinically important. The patients showed, however, statistically significantly improved average scores at admission for moving, sleeping and discomfort and symptoms compared with the time when placed on the waiting list. This is somewhat paradoxical and may reflect patients' expectations on the coming surgical intervention that is supposed to relief the disabling symptoms and to improve function.

Multivariate analysis found that baseline HRQoL and BMI were associated with HRQoL at admission. An increased BMI was associated with a poorer HRQoL and better HRQoL at the time of listing for surgery predicted a better HRQoL at admission. We found, however, no association between the length of waiting time and HRQoL at admission. This result is partially in line with the studies [14-17,27] that have found no significant differences in HRQoL between patients with short waits and those with longer waits. The explanations are various and should be analysed in more detail. For example, it might be possible that after making a decision to operate, the certainty of treatment has a positive impact on health status. Nilsdotter et al. [15] have talked about "regression to the mean", in that with the decision, the health status may even improve. In addition, Achat et al. [29] have found that optimism in older patients is associated with better general health perception. Although patients' HRQoL did not seem to decrease while waiting and no association between waiting time and poorer HRQoL at admission was found, this does not, however, affect our general conclusion that patients awaiting major joint replacement due to OA suffer from discomfort and symptoms, and have a clear reduction in moving, usual activities, sleeping, energy, sexual life and some mental aspects (distress, depression). Although further deterioration in HRQoL may be limited after placement on the waiting list, delayed access to surgery impose the burden of disease.

There were some limitations in our study. First, most patients were residing in the urban area, which may limit our study's generalizability to rural populations. A previous study has shown that urban THR patients may differ from rural patients with respect to pain threshold and perceptions on function [30]. Second, the median length of waiting time among patients was rather short (72 days) and thus the sample may have under-represented those having to wait longer and resulted in an underestimation of the waiting time effect on HRQoL. As the median waiting times in Finland are longer, the study's finding should not necessarily be generalised to all patients awaiting THR or TKR. Further, we measured the time between placement on the waiting list and hospital admission instead of following patients from general practitioner's consultation to treatment. Ideally, the whole waiting time from initial referral to the specialist should be monitored [31]. In prospective studies, it is, however, difficult to collect waiting time data through the care process from primary care consultation to treatment. Third, the population controls had more often a professional education compared to the patients, which may have impacted on the findings as socioeconomic status (SES) has been shown to be associated with health status [30,32].

## Conclusion

In these analyses, we found that the length of waiting was unrelated to the poorer HRQoL at admission. Further, moving, sleeping and discomfort and symptoms improved while waiting for surgery. An interesting view concerning these dimensions is that we do not know the association of disease specific medication with HRQoL and reduction in pain during the waiting time. Although patients' HRQoL measured by the generic 15D instrument improved minimally while waiting, a consistently worse HRQoL was observed in patients waiting for major joint replacement compared with population controls. Thus, it is essential to identify on the waiting list those in the poorest health.

## Competing interests

The author(s) declare that they have no competing interests.

## Authors' contributions

JH was the correspondence author of the manuscript and responsible for the integrity of the work as a whole. She contributed as a principal researcher and writer including drafting the article and the analysis and interpretation of data. MB was the leader of the research project. She made contributions to conception and design, acquisition and interpretation of data and participated in the writing process by commenting the manuscript. UT made contributions to design, acquisition, and interpretation of data. HS and PR contributed as specialists in the field, were involved in the design of the study and hypothesis formation and revised the manuscript. SS, ML, PP, KH contributed as specialists in the field of orthopaedic surgery. They made contributions to design and acquisition of data and revised the manuscript. All authors read and approved the final manuscript.
